# Vanishing Lung Syndrome in a Young Male With Chronic Marijuana Use: A Case Report

**DOI:** 10.7759/cureus.51223

**Published:** 2023-12-28

**Authors:** Gabriel Velez Oquendo, Nivedha Balaji, Aleksandra Ignatowicz, Hisham Qutob

**Affiliations:** 1 Internal Medicine, Northeast Georgia Medical Center Gainsville, Gainesville, USA; 2 Critical Care, Northeast Georgia Medical Center Gainsville, Gainesville, USA

**Keywords:** chronic cough, cough, vanishing lung, marijuana use, marijuana, bullous emphysema, emphysema

## Abstract

Vanishing lung syndrome (VLS) also known as type I bullae disease or idiopathic bullous disease is characterized by giant emphysematous bullae that commonly develop in the upper lobes, occupying at least one-third of a hemithorax. It is a progressive and irreversible condition that involves pulmonary parenchymal destruction and alveolar dilation. It is commonly associated with middle-aged tobacco smokers, habitual marijuana users, and those with alpha-1-antitrypsin deficiency. This case involves an incarcerated male in his 30s with chronic marijuana smoking who presented with a three-month history of right-sided chest pain accompanied by cough, hemoptysis, fever, and weight loss. The patient reported month-long atypical chest discomfort associated with a cough productive of bloody sputum and was brought to the ED after developing acutely worsening right-sided chest pain. The patient underwent a chest X-ray that revealed a large pneumothorax on the left. Subsequently, CT chest imaging showed extensive bilateral bullous disease, left upper lobe consolidation, and enlarged mediastinal lymph nodes.

This case illustrates a rare presentation of VLS in the setting of a young patient who other than reported regular marijuana use had no other risk factors and a negative workup for possible etiologies that could cause his severe bullous emphysema, including alpha-1 antitrypsin, HIV, Sjogren's syndrome, pulmonary Langerhans cell histiocytosis, two sputum Mycobacterium tuberculosis tests, and acid-fast bacteria sputum cultures, which were all negative. Identifying and assessing the degree of disease early in this progressive disease helps guide treatment while preventing further deterioration of lung parenchyma.

## Introduction

This case describes a rare cause of emphysematous lung disease known as vanishing lung syndrome (VLS), a type I bullous emphysema or idiopathic bullous disease characterized by irreversible lung parenchymal damage resulting in giant bullous emphysema involving one or both lungs [1,2]. It is characterized as a progressive lung disease that can present with dyspnea, chest tightness, and respiratory failure due to inadequate participation in gas exchange [2,3]. Progression of the disease can lead to pneumothorax, subcutaneous emphysema, and respiratory failure with hypoxia and hypercapnia. The most common factors known to cause the development of VLS include alpha-1 antitrypsin deficiency, human immunodeficiency virus (HIV), Ehlers-Danlos syndrome, Marfan syndrome, tobacco smoking, marijuana smoking, and sarcoidosis [2,4]. Studies have shown that there are higher rates of emphysematous disease among marijuana smokers when compared to non-smokers and tobacco-only smokers [3]. High-resolution computed tomography scan (HRCT) remains the standard modality for diagnosis of VLS and its complications; with imaging demonstrating asymmetric subpleural bullae located unilaterally and predominately affecting the upper lobes, as was seen in this case [2,4].

## Case presentation

**Figure 1:**
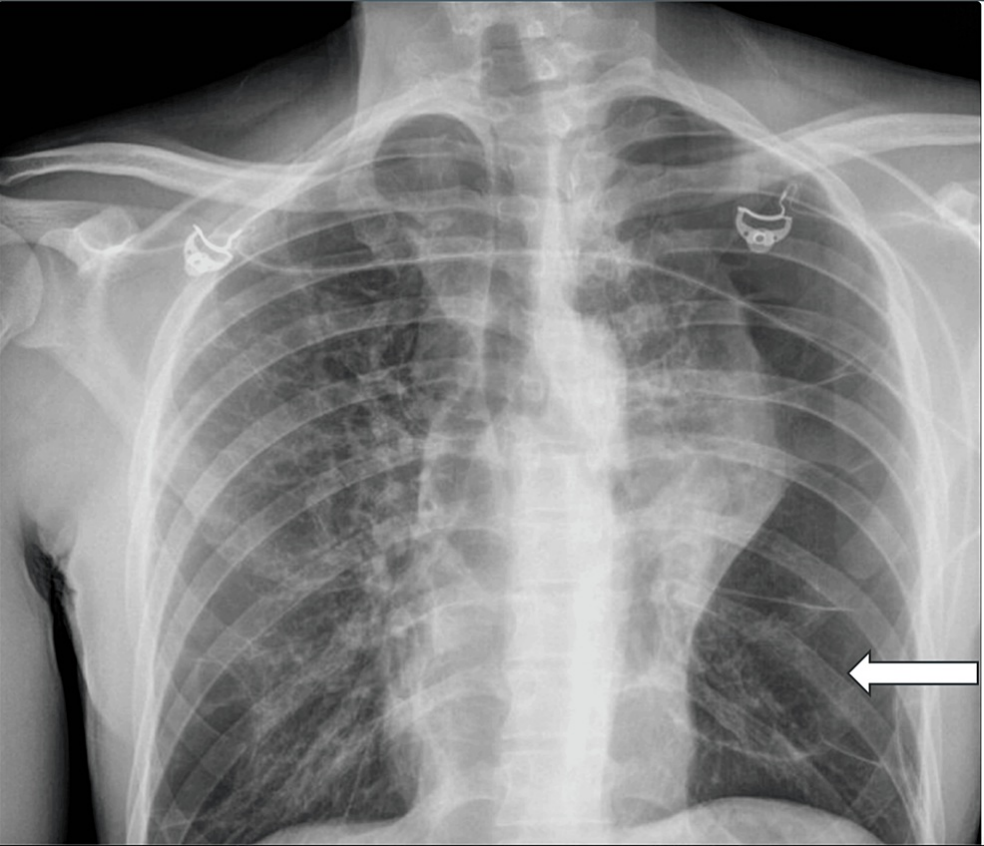
Portable chest radiography during admission showing marked lucency at the lateral aspect of the left lung.

**Figure 2:**
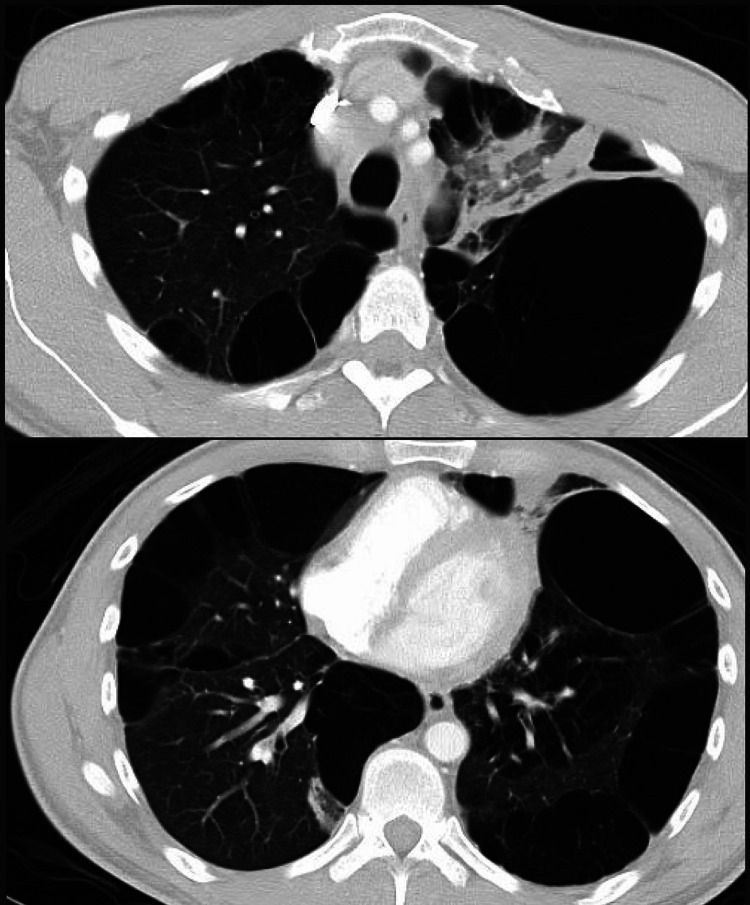
Computed tomography of the chest showing extensive bullous changes in the lungs bilaterally most prevalent in the left lower lobe. Extensive pleural-based consolidation in the left upper lobe extending from the hilum representing possible obstructive changes.

A 34-year-old incarcerated male with a past medical history significant for daily marijuana smoking for 20 years and intermittent tobacco use for four years presented to the emergency department due to worsening left-sided chest discomfort associated with fever, cough, hemoptysis, and weight loss of two months duration. On presentation, the exam was notable a height of 5' 11" and a weight of 131 lbs, a blood pressure of 102/81 mmHg, tachycardia with a heart rate of 103-104 beats/min, and tachypnea with a respiratory rate of 20 breaths/min. On exam, the patient complained of chest pain, dyspnea on exertion, chest tightness, and generalized weakness; pulmonary effort and lung sounds were normal. Laboratory workup with initial CBC showed a WBC count of 11.0 K/uL, hemoglobin of 13.0 g/dL, hematocrit of 39.8%, and 0% bands. Chemistry showed hyponatremia with sodium of 133 mmol/L, hypochloremia with chloride of 98 mmol/L, and elevated creatinine of 1.33 mg/dL. High sensitivity troponin I was normal with a level of 21 ng/L. Subsequently, a chest X-ray was performed, which showed a marked lucency at the lateral aspect of the left lung and emphysematous changes in the right lower lung (Figure 1). CT imaging of the chest with contrast was notable for a mass-like consolidation in the left upper lobe extending from the hilum, and extensive bullous emphysematous changes bilaterally (Figure 2).

In the setting of imaging showing a mass-like consolidation, the patient was started on community-acquired pneumonia empiric antibiotic coverage with azithromycin 10 mg/kg on day one followed by 5 mg/kg daily and ceftriaxone 1g daily for a total of seven days. This treatment was associated with pain management and nebulized medications including albuterol and ipratropium. An extensive workup was started to confirm or rule out an infectious etiology of the patient's presenting symptoms. This workup included a collection of blood cultures, sputum cultures, and left upper lobe mass CT-guided biopsy performed by interventional radiology to get a direct specimen of the pleural base consolidation in the left upper lobe.

The pulmonology service was consulted for further evaluation of the extensive bullous emphysema noted in this young male. A bronchoscopy was performed to evaluate the patient's emphysematous disease and to perform an endobronchial ultrasound-guided (EBUS) biopsy to further investigate the left upper lobe mass abnormality noted on CT of the chest imaging. On bronchoscopy, there was no endobronchial abnormality in the trachea, right mainstem, right upper, middle, and lower lobe. In the left mainstem, there was friable tissue with airway bleeding, and an endobronchial lesion completely opacifying the left upper lobe orifice. At the subcarinal area, there was thickened tissue with neoplastic mucosa underneath consistent with a neoplastic process that was confirmed after a biopsy of the tissue reported poorly differentiated adenocarcinoma of the lung.

Moreover, the patient completed the antibiotic regimen for what was thought to be community-acquired pneumonia, but due to the rare presentation and significant emphysematous disease in this young male, conditions, and diseases that were considered as possible etiologies for this patient’s severe bullous emphysema were tested including alpha-1 antitrypsin, human immunodeficiency virus, Sjogren's syndrome associated lymphocytic interstitial pneumonia, pulmonary Langerhans cell histiocytosis, Birt-Hogg-Dube syndrome, Marfan syndrome, lymphocytic interstitial pneumonia, and emphysema. Lymphangioleiomyomatosis was not considered due to its presentation being exceedingly rare in men, especially in patients without tuberous sclerosis and imaging showing cysts that do not fit the clinical pattern on this patient’s computed tomography scan. Although the patient’s Sjogren antibody was positive, clinically he was not presenting active Sjogren's disease symptoms. The patient had lab work conducted for the other above-mentioned disease processes, but all resulted negatively (Table 1).

**Table 1:** Autoimmune testing

Tests	a1-antitrypsin	HIV 1/2	Scl 70 Ab, IgG, S	Sjogren's Antibody (SS-A)	ANA RFLX to Auto ABS	Sjogren's Antibody SS-B	Sm Ab, IgG	RNP Ab, IgG	Jo 1 Ab, IgG, S	DNA Double-stranded Ab, IgG	Centro-mere IgG
Range	90-200 mg/dL	Nonreactive	<1.0 (negative)	<0.1 (negative)	Negative	<1.0 (Negative) U	<1.0 (Negative) U	<1.0 (Negative) U	<1.0 (Negative) U	<30.0 (Negative) IU/mL	<1.0 (Negative) U
Result	218 mg/dL	Nonreactive	<0.2	>8.0	Positive	<0.2	<0.2	<0.2	<0.2	<12.3	<0.2

**Figure 3:**
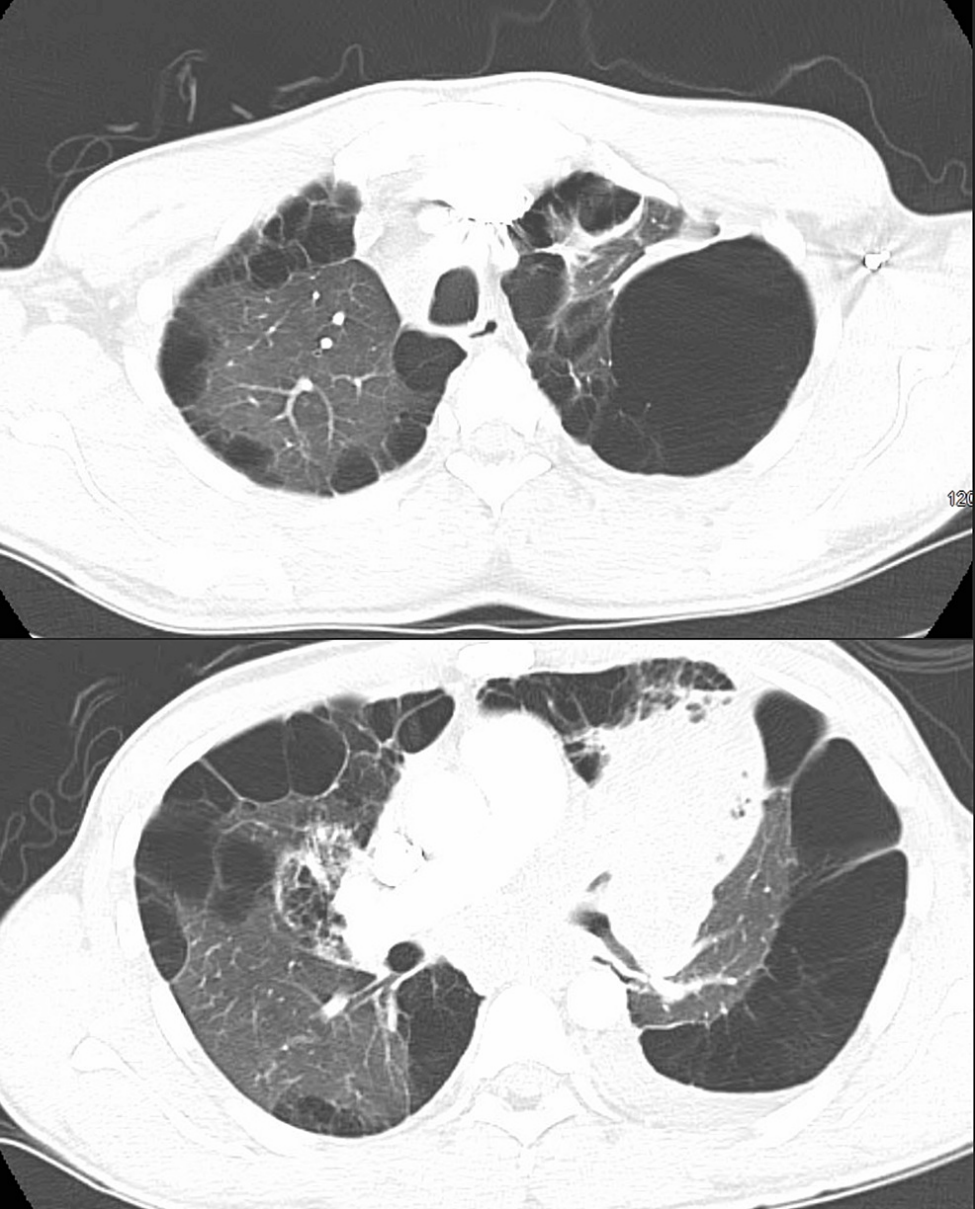
Pulmonary CTA performed due to suspicion of pulmonary embolism did not show evidence of pulmonary embolism, no significant interval change in the size of the large left upper lobe mass and associated consolidation, and severe bilateral bullous emphysema. CTA: computed tomography angiography

Unfortunately, during the hospital course, the patient suddenly developed tachycardia, tachypnea, and hypotension which prompted the team to calculate a Wells score for his risk of pulmonary embolism due to the patient's known presence of malignancy diagnosed after EBUS biopsy. The calculated Wells score was 4.0 points which translated to a moderate risk group with a 16% chance of having a pulmonary embolism; with subsequent d-dimer testing reporting a level of >4.00. A CTA pulmonary was subsequently performed which reported no evidence of a pulmonary embolism, no significant interval changes in the size of the large left upper lobe mass, and unchanged severe bilateral bullous emphysema (Figure 3).

At the end of the hospitalization, the patient was diagnosed with type I bullous emphysema also known as VLS. Unfortunately, the patient followed hematology and oncology for a few months for treatment of adenocarcinoma of the lung before being lost to follow-up.

## Discussion

VLS or type I bullous emphysema is a condition that most commonly affects people over age 45 with concomitant tobacco smoking or at an earlier age in patients with a history of alpha-1 antitrypsin deficiency and/or marijuana use [2]. Cannabis is the most widely used illicit substance in the world and the second most smoked substance after tobacco products, with emphysematous disease being more prevalent amongst marijuana smokers compared to non-smokers and tobacco-only smokers [1]. Chronic marijuana smoking can lead to parenchymal and histologic changes because of airway inflammation and irritation secondary to prolonged inhalation of toxic particles and elevated inspiratory pressures from deep inhalation. Histopathologic studies have demonstrated extensive goblet cell hyperplasia, blunting of ciliated epithelial cells, intra-alveolar accumulation of pigmented histiocytes, and extensive inflammation of the intra-epithelial and subepithelial cells [1,2]. These changes can be seen as poorly defined centrilobular ground-glass opacities on imaging and can be differentiated between tobacco-induced bullae which are typically centrilobular and uniformly distributed while marijuana-induced bullous emphysema is situated in a pre-septal distribution along the upper lobes [5]. It is thought that the destruction of the alveolar walls from smoking occurs due to deep inhalation, long breath-holding time, and high inspiratory pressures, this results in subpleural blebs that can gradually merge to form larger bullae and diffuse emphysematous changes from elastase release, causing major complications including the formation of pneumothorax [6-10].

Radiographic diagnostic criteria for type I bullous emphysema include the presence of giant bullae in one or both upper lobes, occupying at least one-third of the hemithorax, and compressing surrounding normal lung parenchyma [1-4,10,11]. Diagnostic work-up most commonly includes pulmonary function testing, arterial blood gas, and serum alpha-1 antitrypsin level [4,9]. HRCT is used to assess lung parenchyma and determine complications and coexisting conditions of VLS, which include pneumothorax, subcutaneous emphysema, bronchiectasis, pulmonary arterial enlargement, and infected cyst which can further worsen respiratory failure and promote clinical deterioration [2,10,11].

Although the patient had a positive Sjogren antibody, there were no pulmonary manifestations suggestive of Sjogren’s syndrome such as dry cough, airway lesions, imaging without findings of ground glass opacities, and non-septal linear opacities [12,13]. Moreover, the patient did not have sicca symptoms, or arthralgias, and bronchoalveolar lavage was negative for lymphocytic alveolitis [13].

Unfortunately, conservative methods such as nebulized bronchodilators are mostly used in asymptomatic patients, with surgical resection being recommended to patients that have large symptomatic bullae and secondary complications such as the development of pneumothorax [4]. Surgical methods include stapled bullectomy, endocavitary drainage, video-guided thoracoscopic volume reduction surgery, one-way endobronchial valve placement, and lung transplantation. Surgical resection can help reduce dynamic lung hyperinflation, decrease the risk of pneumothorax, and improve dyspnea, pulmonary function, exercise performance, and quality of life, but it has not shown mortality benefit [2,4,5]. On average, patients will experience improvements in daily function from surgical interventions for about four years before experiencing a functional decline [11]. When surgical resection is not an option and the patient experiences a further respiratory decline, palliative medicine should be considered due to poor prognosis [10].

Furthermore, emphysema is an independent risk factor for lung cancer (LC). This risk increases with higher rates of smoking. Pulmonary areas with greater CT-assessed emphysema severity are associated with the highest risk of developing LC. The most frequent histological lineage in patients with emphysema and LC is adenocarcinoma. Lastly, there are multiple potential pathways implicated in this association, though in all cases through chronic inflammatory status and anomalous cellular repair, which, along with polymorphisms in various genes, serve to trigger carcinogenesis [13,14].

Our case report highlights the development of severe bullous disease in a relatively young patient, which likely can be attributed to his chronic marijuana smoking. While the pathophysiology of VLS is still unclear, there is concern that marijuana smoking products can act as catalysts for the extensive parenchymal changes that occur in VLS. Additionally, the legalization of marijuana globally for both recreational and medicinal purposes strengthens the need for increased awareness of its deleterious effects. In addition to the increased risk of developing a chronic cough, bronchitis, and malignancy, patients should also be counseled on the risk of developing VLS. Physicians should have a low threshold to screen patients already exposed to marijuana and tobacco products.

## Conclusions

This case highlights the importance of thorough history taking despite no family history, detailed physical examination, and skilled radiography interpretation to diagnose type I bullous emphysema or VLS. Timely diagnosis is essential to providing proper management and preventing its progression into severe emphysematous disease. These patients should be strongly counseled and encouraged on tobacco and marijuana cessation due to the detrimental effects on lung parenchyma. Although emphysema has been shown to increase the risk of lung malignancy, marijuana use has not been documented in the literature as a risk factor, but it is possible that preventative LC screening guidelines for chronic tobacco use are of benefit to patients with chronic marijuana use. Furthermore, patients should be referred to pulmonology and cardiothoracic surgery to further delineate patients deemed for surgical resection, conservative management, or palliation.
